# Improving the Mental Health of Nursing Staff Seen from the Perspective of Staff a Preliminary Study

**DOI:** 10.3390/medicina61091573

**Published:** 2025-08-31

**Authors:** Rudina Çerçizaj, Fatjona Kamberi, Emirjona Kiçaj, Vasilika Prifti, Sonila Qirko, Erlini Kokalla, Liliana Rogozea

**Affiliations:** 1 Faculty of Medicine, Transylvania University of Brasov, 500036 Brasov, Romania; emirjona.kicaj@unitbv.ro (E.K.); vasilika.prifti@unitbv.ro (V.P.); sonila.qirko@unitbv.ro (S.Q.); r_liliana@unitbv.ro (L.R.); 2 Faculty of Health, University of Vlora “Ismail Qemali”, 9401 Vlore, Albania; fatjona.kamberi@univlora.edu.al (F.K.); erlini.kokalla@univlora.edu.al (E.K.)

**Keywords:** mental health of nurses, COVID-19, coping strategies, institutional support, reflective analysis and SWOT

## Abstract

*Background*: During the COVID-19 pandemic, nurses faced enormous emotional challenges and profound physical fatigue, as well as constant concerns about whether they would receive genuine support in the workplace. *Objectives*: This study aimed to assess the long-term impact of the COVID-19 pandemic on the mental health of nurses and to identify key challenges, coping strategies and needs for institutional support. *Methods*: It was conducted in Albania and followed a mixed-methods design in two interconnected phases, using a mixed-methods approach. Phase I included a quantitative survey of 288 nurses from regional hospitals in Fier and Vlora using a structured questionnaire covering five domains: workplace challenges, stress and work–life balance, health effects and burnout, coping mechanisms, and suggestions for improvement. Data were analyzed using descriptive statistics. Phase II consisted of a reflective seminar with 47 nurses selected from the initial sample. Participants shared their post-pandemic experiences, coping strategies, and proposals for improving mental well-being. SWOT analysis was used to structure the reflections and identify internal and external factors influencing nurses’ mental health. *Results*: The results showed that nurses continue to face high workload, insufficient psychological support, and that 37.5% reported their work–life balance had worsened since the pandemic (21.9% sometimes; 15.6% most of the time). Participation in the reflective seminar had a positive impact on increasing professional awareness. *Conclusions*: These findings highlight the need for structured and sustainable interventions within healthcare institutions to protect and promote nurses’ mental health in post-crisis contexts.

## 1. Introduction

The COVID-19 pandemic has caused an extraordinary crisis in global health systems, placing nurses on the front lines of the fight against the virus. International studies have found that nurses experienced high levels of stress, anxiety, and depression during the pandemic period due to high workload, lack of resources, and fear of infection [1,2,3,4,5,6]. In Albania, where staff shortages and limited infrastructure challenge the health system, the impact of the pandemic on nurses’ mental health has been equally severe. Throughout the waves of the COVID-19 pandemic, many Albanian nurses endured exhausting workloads. Meanwhile, they werestruggling without psychological support, becoming emotionally drained, increasingly vulnerable to burnout, and deeply affected in their mental and emotional well-being [7].The international literature has identified key challenges affecting the mental health of nurses, including high workload, staff shortages, inadequate working conditions, and lack of psychological support [2,8]. In resource-limited settings, these challenges are exacerbated. Regional studies from the Balkans highlight that many nurses not only struggle with demanding working conditions but also face the weight of social stigma and a lack of access to training that could help them cope with stress. These ongoing challenges can slowly diminish their motivation and daily performance but may also hinder their ability to provide the compassionate, high-quality care that patients deserve [9]. Studying the mental health of nurses from their perspective is crucial to understanding the daily realities experienced in clinical settings and their impact on psychological well-being. Nurses themselves are the most direct witnesses tothe emotional burden, work pressure, and lack of structural support. Therefore, their voices represent a valuable resource for designing effective interventions.

During the COVID-19 pandemic, frontline nurses faced enormous emotional challenges and profound physical fatigue, as well as constant concerns about whether they would receive genuine support in the workplace. These difficulties profoundly affected nurses’ mental health and were directly reflected in the quality of care they could provide to patients [3]. Moreover, many qualitative studies highlight the importance of listening to the personal experiences of nurses who report constant fatigue, anxiety, and isolation, in order to better understand the challenges they face every day. These experiences often reveal deeper systematic problems, such as unfair workloads and lack of control over their professional lives and careers [4]. For example, a study of 579 Finnish nurses revealed that many of them sought more personalized support from their employers, including recognition of their efforts, safer working conditions, open and honest communication, and strong leadership during crises [10]. A systematic review of 28 studies involving more than 1100 nurses from different countries found that psychological and social support is vital. This form of care is essential not only to help nurses develop resilience in the face of adversity but also to maintain their mental and emotional balance in an often tiring and challenging work environment [11].

This study employed a cross-sectional research design with a mixed-methods approach, conducted in two clearly defined and interrelated phases. The aim was to assess the professional, organizational, and psychological challenges faced by nurses after the COVID-19 pandemic and to identify strategies to improve their mental health and well-being in the workplace.

Research questions:

1. What are the main challenges that have affected the mental health of nurses in Albania after the COVID-19 pandemic?
2. What are the most common ways of coping with stress used by nurses in Albania?
3. What are the suggestions and recommendations of nurses in Albania themselves for improving mental health in the workplace?

## 2. Materials and Methods

### 2.1. Study Design

This preliminary exploratory study employed a mixed-methods design with two clearly defined and interrelated phases, aiming to assess the professional, organizational, and psychological challenges faced by nurses following the COVID-19 pandemic and identify strategies to enhance their mental health and well-being in the workplace.

Phase I involved a quantitative survey administered through a structured questionnaire to systematically capture key post-pandemic challenges, coping mechanisms, and improvement suggestions for nurses. Data from this phase were analyzed using descriptive statistics (frequencies and percentages) to provide a clear distribution of responses across all items.

Phase II involved a reflective seminar—designed as an educational, discussions-based intervention building directly upon the findings from Phase I. This phase aimed to promote awareness, encourage critical reflection, and facilitate the co-creation of action strategies with participants.

### 2.2. Study Settings

The study is part of a larger study that aims to analyze the impact of the COVID-19 pandemic on the mental health of nurses. In a previous phase, nurses’ perceptions of their mental health and well-being were assessed and analyzed using two standardized and validated instruments: the DASS-21 and the Personal Assessment: 8 Dimensions of Wellness. The data were collected during and after the pandemic period and also included the main challenges that nurses have encountered in the post-COVID period. These findings were used to design the present two-phase study, where the second phase (reflective seminar) was planned from the outset as an in-depth follow-up to the first phase.

Phase I—A structured questionnaire, specifically designed for this study and containing closed-ended questions, rated on three-point scale, was administered to nurses from public health institutions in the cities of Fier and Vlora.

Participants were recruited using purposive sampling, targeting only those nurses who had been directly involved in patient care during the pandemic.They ensured that the sample represented professionals with first-hand experience of pandemic-related challenges.

The questionnaire includes five main sections: (1) weaknesses in the workplace, (2) the impact of ongoing stress after the pandemic, (3) health effects and burnout, (4) coping mechanisms and self-care, and (5) suggestions and perceptions for the future. Data from this phase were analyzed through descriptive statistics to identify the distribution and percentages of responses in each category.

Phase II—Following Phase I, a reflective seminar was organized with selected nurses from the initial sample to explore the identified challenges in greater depth and to collect experience-based recommendations.

The seminar, titled “Challenges and Opportunities for the Nursing Profession after COVID-19” was held on 23 November 2024, at the Faculty of Health, University “Ismail Qemali”of Vlora [12]. It was accredited by the Albanian National Center for Continuing Education (ASCK) and awarded four continuing education credits. In addition to serving as a qualitative data collection tool, the seminar was explicitly designed as an educational intervention to strengthen professional insight and emotional well-being among nurses.

The reflective seminar lasted 4 h and 30 min and included presentations, group reflection, discussions, and pre- and post-intervention testing. It was structured to promote professional awareness, identify challenges, and discuss practical strategies for improving mental health and rebuilding the nursing profession in the post-pandemicperiod.

### 2.3. Study Sample

A total of 288 nurses from public health institutions in the cities of Fier and Vlora participated in Phase I.The sampling was purposive, and inclusion required involvementin patient care during the COVID-19 pandemic. Participation was voluntary and anonymous to ensure a safe environment for sharing experiences.

Reflective seminar care inclusion criteria (Phase II):

Forty-seven nurses were selected from the initial sample based on three criteria:

1. Direct pandemic care experience—nurses had been directly involved in patient care during the COVID-19 pandemic.
2. Willingness to engage—expressed readiness to participate in reflective discussions on post-pandemic challenges.
3. Significant challenges—had reported substantial professional or psychological challenges in Phase I.

Selection also aimed to ensure role diversity (representation from different hospital departments, various years of professional experience, and different shift patterns).

Recruitment continued until informational saturation was reached, meaning no new themes emerged from participants’ contributions.

### 2.4. Data Sources/Measurement

#### 2.4.1. Data Sources During the First Phase

Data for Phase I were collected through a structured questionnaire specifically designed for this study. The questionnaire contained closed questions with three response options: “Rarely,” “Sometimes”, “Most of the time.” It was divided into five thematic sections, which addressed the most critical areas related to the experience of nurses after the pandemic:

- Vulnerabilities in the workplace.
- Stress and work–life balance.
- Health effects and burnout.
- Coping mechanisms and self-care.
- Views and suggestions for the future.

#### 2.4.2. Data Sources During the Second Phase

In Phase II, qualitative data were collected during a reflective seminar with 47 nurses selected from the Phase I sample. This seminar was explicitly planned from the outset as the second phase of the study and served both as a qualitative data collection tool and as an educational intervention to strengthen professional insight and emotional well-being among nurses.

During the seminar, participants completed an open-ended reflective questionnaire, which included the following prompts.

- What challenges most affected your mental health after the pandemic?
- What coping mechanisms or strategies helped you the most?
- What support did you expect from the institution but did not receive?
- What concrete recommendations do you have to improve mental well-being?
- What training or programs would be helpful for you?

In addition, participants completed an institutional program evaluation form assessing the availability of 12 support interventions, including:

Resilience training

Cross-department training

Mental health improvement programs

Leadership development

Crisis training

Diversity training

Ongoing professional training

New care models

Empathic care practices

Unit capacity management

Pediatric care plans for critical situations,

Other (e.g., psychological support groups)

#### 2.4.3. Questionnaire Distribution and Data Collection

A total of 300 questionnaires were distributed to nurses employed at the Fier and Vlora Regional Hospitals. The distribution targeted professionals who had been directly involved in patient care during the COVID-19 pandemic and who were at work at the time of data collection.

Participants were selected through purposive sampling to include only those with direct pandemic care experience and who agreed to participate. Data collectionwas coordinated in person by trained researchers or institutional coordinators, who explained the study and assured confidentiality. Questionnaires were distributed directly to the nurses; most participants completed the questionnaires immediately on site, whereas a smaller number returned them within a few days. Of the 300 questionnaires distributed, 288 were returned completed and valid for analysis, representing a very high participation rate of 96%.

The reflective seminar in Phase II included the administration of the open-ended questionnaire and the institutional support evaluation. Responses from the instruments were analyzed using a SWOT framework, categorizing factors into strengths, weaknesses, opportunities, and threats affecting nursing mental health in the post-pandemic period. This structured approach allowed for the identification of gaps and opportunities for sustainable interventions at personal and institutional levels.

#### 2.4.4. Pilot Study

Before distibuting the large-scale questionnaire, a pilot test was conducted with a group of nurses (*n* = 20) who met the study inclusion criteria. The objectives of the pilot phase was to assess the clarity of the questions, the clarity and comprehensibility of questions, and the appropriateness of the wording and the time required for completion. Participants were asked to provide feedback on the structure, wording and overall usability of the instrument. Based on their input, minor wording modifications wee made to improve clarity and ensure content validity. The final revised version of the questionnaire was then used for data collection in Phase I of the study.

### 2.5. Data Collection and Analysis

The data were collected in two clear phases.

Phase I—A structured study,-specific questionnaire with closed - ended items rated on a three- point scale of “Rarely”, “Sometimes”, and “Most of the time” was administered to 288 nurses. Descriptive statistics (frequencies and percentages) were calculated to identify the most frecuently reported post-pandemic challenges.

Phase II—A reflective seminar with 47 nurses selected from Phase I participants was conducted to deepen the exploration of key challenges and gather practical recommendations. Inclusion criteria for Phase II participants included: (1) direct involvement in patient care during the COVID-19 pandemic, (2) willingness to participate in reflective discussions, and (3) reporting of significant challenges in Phase I.

Nurses completed:

- A pre- and post-intervention reflective questionnaire with prompts (e.g., main challenges, coping strategies, gaps in institutional support, suggestions for improvement).
- An institutional program evaluation form assessing the availability of 12 potential support interventions.

**Figure 1:**
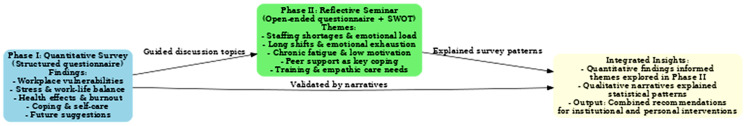
Integration of quantitative and qualitative Findings.

The qualitative data was analyzed through thematic analysis, structured using a SWOT framework, to identify both internal and external factors influencing post-pandemic nurse mental health. Integration of the datasets was performed by mapping quantitative findings from Phase I to qualitative themes from Phase II, allowing for the triangulation and validation of results (Figure 1).

#### 2.5.1. Measured Variables

In Phase I, data were collected using a structured questionnaire specifically designed for this study consisting of closed-ended questions rated on three-point scale (“Rarely”, “Sometimes”, “Most of the time”). The instrument measured five key domains, each with a specific number of items and an example question:

1. Challenges and difficulties in the workplace—four items (e.g., staff shortages, overload and constant pressure, insufficient communication and support).
2. Impact of work on personal life and work–life balance—three items (e.g., continuous stress, deterioration in work–life balance, and need for flexible work policies).
3. Effect on physical and mental health—three items (e.g., burnout, insufficient psychological support, and need for workplace wellness programs).
4. Coping strategies and self-care—three items (e.g., physical activity and recreation, support from colleagues, and health maintenance through leisure policies).
5. Suggestions and improving working conditions and institutional support— three items (improving working conditions, investment in professional training, development of psychological support policies).

In total, the questionnaire comprised 16 items, with each domain containing between three and four questions (Table 1 for full item wording). Higher scores indicated greater frequency or perceived impact.

In Phase II, a pre- and post-intervention reflective questionnaire was administered in paper format during the seminar to assess changes in participant’s perceptions and self-reported awareness following the group discussions. The instrument included eight closed-ended items scored on three-point scale, plus open-ended prompts designed to elicit deeper insight into:

▪ Key post-pandemic challenges;
▪ Effective individual coping strategies;
▪ Perceived gaps in institutional support;
▪ Concrete suggestions for improvement.

An example closed-ended item from this questionnaire is: I feel more confident in identifying strategies to support my mental well-being at work.

Qualitative responses from the open-ended section were analyzed using a SWOT framework (Strengths, Weaknesses, Opportunities, and Threats) to identify internal and external factors influencing nurses’ mental health at both personal and institutional levels.

#### 2.5.2. Statistical Methods

The quantitative data from Phase I were analyzed using IBM SPSS Statistics version 26.0 (IBM Corp, Armonk, NY, USA). Descriptive statistics (frequencies and percentages) were calculated for each question and section to identify the most common challenges experienced by nurses in the post-pandemic period.

Findings from Phase I informed the design of Phase II, which consisted of intervention through a reflective training seminar. This phase included group-based reflective activities using three instruments.

- A structured open-ended reflection questionnaire;
- A program evaluation form assessing institutional support and provided training;
- A SWOT analysis worksheet.

The qualitative data from the open-ended reflection questionnaire and the qualitative section of the program evaluation form were analyzed in an integrated manner through the SWOT framework. This method allowed for the systematic identification of both internal factors (strengths and weaknesses) and external factors (opportunities and threats) affecting nurses’ mental health at personal and institutional levels.

### 2.6. Ethical Considerations

The study received ethical approval from the Research Ethics Committee at the Faculty of Health, University of Vlore “Ismail Qemali,” Albania, (Decision No. 80, dated 13 March 2024). All participants provided informed consent and were assured of anonymity and confidentiality. Data were used solely for research purposes, in accordance with established ethical standards for research involving human subjects.

## 3. Results

Phase I—Qualitative survey: Challenges, coping mechanisms, and suggestions.

The first phase of the study analyzed the responses of 288 nurses, who completed a structured questionnaire on the challenges experienced in the period following the COVID-19 pandemic. The most common challenges were staff shortages, high workload, emotional pressure, andreports of worsened work–life balance since the pandemic. Specifically, 62.5% indicated that their work–life balance had rarely worsened since the pandemic, 21.9% reported worsening sometimes, and 15.6% most of the time. Participants also reported significant impacts on mental and physical health, as well as the need for institutional support and specialized training. Individual coping strategies included self-care, support from colleagues, and engagement in personal activities outside of work.

### 3.1. Phase I. Challenges Faced by Nurses After the Pandemic—Coping Mechanisms and Suggestions for the Future

As we can see in Table 1, of the 288 participating nurses, the majority were female (82.1%), with a largest proportion having 6–10 years of experience (28.5%). During the pandemic, 19.1% worked in COVID-19 dedicated wards, while after the pandemic, the largest share (21.9) were in maternity wards. Most of the nurses worked 40 h/week during (52.8) t and after (60.1%) the pandemic, though exceeded hours (>40 h) were reported by 34% during the pandemic and 26.4% after. Notably, 87.8% received special training during COVID-19, indicating strong engagement in professional development during the crisis.

In Table 2, the most frequently reported challenges were staff shortages leading to unstable workloads, insufficient communication/support, and the need for improved infrastructure, technology, and training. While most of the nurses reported these issues rarely, 21.5% experienced high workloads sometimes, and 11.5%most of the time. Continuous stress was reported most of the time in 14.2% of the participants. Regarding work–life balance, 62.5% indicated that it had rarely worsened since the pandemic, 21.9% reported worsening sometimes, and 15.6% most of the time. Burnout or mental health issues were reported sometimes by 23.2% of participants and most of the time by 11.11%. Psychological support from institutions was reported as insufficient by 70.1%.

The most common coping mechanisms included physical/recreational activities (reported most of the time by 16.7% of nurses), and 20.5% indicated that improving working conditions isimportant most of the time, while 87.8% reported that their institution rarely prioritizes psychological support policies. Investment in professional training was considered necessary most of the time by only 1.7% of participants (Table 3).

### 3.2. Phase II. Reflective Training Seminar and Qualitative Analysis

Phase II—Reflective training seminar and qualitative analysis

Phase II—Educational and reflective training seminar developed from Phase I findings. This intervention included:

1. Group-based reflective seminar activities using a structured open-ended questionnaire, program evaluation form, and SWOT analysis worksheet.
2. A pre- and post-seminar closed-ended questionnaire to measure short-term changes in knowledge and awareness.

Most participants were female (80.9%) with an average age of 34 years (±3.4). Over halfof the participants (59.6%) were from the Regional Hospital of Vlora, while the remaining 40.4%were from the Regional Hospital of Fier (Table 4).

#### Interpretation of SWOT Analysis Results

SWOT analysis was used as a qualitative tool to structure the reflections of 47 nurses participating in the reflective seminar.

Strengths identified included self-care, physical activity, family support, and solidarity among staff, with 48.9% reporting participation in ongoing training. Weaknesses emerged in the form of lack of emotional recovery structures, insufficient breaks, and limited mental health training (19.1%) and resilience (25.5%). Opportunities focused on developing psychological support systems, leadership programs, and stress management initiatives, with 33.3% noting the implementation of new healthcare programs (Table 5).

Threats included chronic burnout, risk of leaving the profession, and reduced patient quality of care, exacerbated by the absence of leadership training (80%) and crisistraining (70.2%).

The results show a clear improvement across all measured areas following the seminar. Notable increases were absorbed in Table 6:

1. Understanding of the impacts on mental health (from 27.7% to 70.2% after);
2. Identification of practical solutions (from 31.9% to 76.6%);
3. Awareness of coping strategies (from 25.5% to 42.6% and from 23.4% to 46.8% for the two related items);
4. Recognition of importance of work–life balance (from 27.7% to 53.2%).

Increases were also recorded in knowledge about current issues (from 29.8% to 63.8%), the contribution to the profession (from 27.7% to 74.5%), and identifying lack of institutional support (from 25.5% to 38.8%).

## 4. Discussion

The findings of this two-phase study provide important insights into the long-term consequences of the COVID-19 pandemic on nurse’s mental health and well-being, and demonstrate how a structured reflective educational intervention can address identified gaps. These results confirm that the pandemic was a significant burden on nurses’ mental health, which is consistent with international evidence on high levels of burnout anxiety and depression among frontline nurses [1,2,4,9]. The discussion below focuses on the key challenges identified, the coping mechanisms used, and the suggestions proposed by nurses to improve their professional environment in the post-pandemic context.

The main challenges faced by nurses since the pandemic included excessive workload, insufficient communication, andlack of institutional support, all of which have been linked to deterioration inwork–life balance and overall well-being [2,9,10].

These stressors of poor conditions and resourse shortages have been strongly associated with burnout [1,2]. Our participants also reported that institutional policies often failed to address thesestressors, reflecting previous findings that healthcare organizations need proactive, long-term strategies to protect staff well-being during and after crises [8,10,12,13].

The primary coping mechanisms used by nurses involved physical and recreational activities, and family and peer support, as reported to the literature [3,4,5,11]. However, the absence of formal emotional recovery structures and limited mental health or resilience training (reported by only 19.1% and 25.5% of participants, respectively) indicates that individual coping was not adequate at the institutional levels, an issue also highlighted in WHO post-pandemic recommendations [14].

Notable findings from the second phase of the study, the reflective training seminar, were related to the reassessment role of nurses during and after the pandemic. Before the seminar, only 23.4% of participants recognized the broader professional value and leadership role of nurses. This increased reflective education can rebuild professional self-esteem and role clarity among nurses [12,15,16,17,18]. The seminar also significantly improved awareness of the mental health impacts of the pandemic (from 27.7% to 95.7%), echoing findings from similar interventions that helped normalize discussions on stress, anxiety, and emotional fatigue among healthcare workers [6,8].

Further positive changes included an increase in knowledge of stress coping strategies (from 25% to 87.2%), and a marked rise in participants willingness to propose concrete workplace solutions (from 31.9% to 95.7%).These outcomes are consistent with studies demonstrating that targeted educational interventions can strengthen resilience, enhance problem-solving skills, and empower nurses to engage in institutional change processes [6,8].

From a broader perspective, our results reinforce that reflection-based educational interventions can serve as an effective, low cost, and scalable strategy to address post-pandemic mental health challenges in nursing. They not only enhance knowledge and awareness but also stimulate active professional engagement in creating healthier work environments. As supported by recent systematic reviews, such interventions can contribute to reducing turnover intentions, improving job satisfaction, and maintaining patient care quality during public health crises [6,8,19,20].

The practical implications of this study include the urgent need for healthcare institutions to invest in continuous mental health and resilience training, implement psychological support systems, and integrate leadership and crisis management programs into routine nursing development [2,8,12,14,20].

Future research should adopt longitudinal designs to evaluate the sustained impact of reflective educational programs on nurses’ mental health and professional performance. Additionally, studies in diverse healthcare settings could explore how institutional culture and resources influence the effectiveness of such interventions.

### Limitations of the Study

This study has several limitations. First, the sample was predominantly female (82.1%), which may limit the applicability of the findings to male nurses. Second, although socio-demographic and professional characteristics such as work experience, weekly working hours, age, marital status, and number of children were collected, their potential influence on the results was not examined in the present analysis. Future research should incorporate these factors to better understand their relationship with mental health outcomes. Third, the study was conducted in only two regional hospitals (Fier and Vlora), which may limit the transferability of findings to other healthcare settings. Fourth, the use of self-report questionnaires may introduce recall and social desirability bias. Fifth, the non-random selection of participants in the reflective phase may limit representativeness. Finally, the absence of a control group and long-term follow-up restricts the ability to assess the sustained impact of the intervention, and the SWOT analysis relies on subjective reflections.

Future research should address these limitations by employing multi-site designs involving diverse healthcare institutions, random or stratified sampling approaches to enhance representativeness, and longitudinal designs to track changes in mental health and well-being over time. Including control groups and integrating both quantitative and qualitative methods across multiple time points would further strengthen causal inferences and the applicability of findings.

## 5. Conclusions

The challenges nurses have faced since the pandemic have had a significant impact on their mental health. High workload, lack of institutional support, and feelings of chronic fatigue were frequently cited as key concerns.

The coping strategies used by nurses are primarily individual, such as physical activity, self-care, and support from colleagues, but are often insufficient without a formal support system. Participants proposed concrete and actionable recommendations, including enhanced training on mental health, the establishment of institutional support mechanisms, and the improvement of working conditions to promote work–life balance. The seminar intervention was perceived as highly beneficial, not only in enhancing knowledge but also in fostering awareness, critical reflection, and collaborative problem-solving. The application of SWOT analysis amplified the nurses’ voices, offering valuable insights into existing strengths, identifying gaps, and highlighting areas for improvement at both personal and institutional levels. These findings underscore the urgent need for targeted, system-level interventions to safeguard nurses’ mental health and strengthen the resilience of the nursing workforce in the post-pandemic era.

## Figures and Tables

**Table 1:** Socio-demographic and professional data of nurses (*n* = 288).

Category	Subcategory	Frequency (*n*)	Percentage (%)
Gender	Male	52	17.9
	Female	236	82.1
Work Experience (Years)	≤5 years	74	25.7
	6–10 years	82	28.5
	11–20 years	57	19.8
	>20 years	75	26.0
Self-Reported Weekly Working Hours During COVID-19	<40 h	38	13.2
	40 h	152	52.8
	>40 h	98	34.0
Weekly Working Hours (Current Period)	<40 h	39	13.5
	40 h	173	60.1
	>40 h	76	26.4
Work Department During COVID-19	Intensive Care Unit (ICU)	11	3.8
	Surgery	22	7.6
	Operating Room	12	4.2
	ENT/Ophthalmology	15	5.2
	Emergency	38	13.2
	Pathology	24	8.3
	COVID-19 Dedicated Ward	55	19.1
	Pediatrics	41	14.2
	Dispensary	4	1.4
	Maternity	52	18.1
	Pediatrics/ICU	14	4.9
Current Work Department	Intensive Care Unit (ICU)	12	4.2
	Surgery	14	4.9
	Infectious Diseases	26	9.0
	Operating Room	15	5.2
	ENT/Ophthalmology	16	5.6
	Emergency	32	11.1
	Pathology	44	15.3
	Pediatrics	45	15.6
	Dispensary	2	0.7
	Maternity	63	21.9
	Imaging	2	0.7
	Pediatrics/ICU	17	5.9
Received Training During COVID-19	Yes	253	87.8
	No	35	12.2

**Table 2:** Challenges faced by nurses after the pandemic.

Challenges Faced After the Pandemic			*n*	%
Workplace Weaknesses	Have you identified major weaknesses in the resources and infrastructure available at your workplace since the pandemic?	Rarely	226	78.5
		Sometimes	57	19.8
		Most of the time	5	1.7
	Are workloads unsustainable due to staff shortages?	Rarely	193	67
		Sometimes	62	21.5
		Most of the time	33	11.5
	Has communication and support from your institution been insufficient after the pandemic?	Rarely	187	64.9
		Sometimes	59	20.5
		Most of the time	41	14.2
	Does your institution need to improve technological resources and staff training to address post-pandemic challenges?	Rarely	227	78.8
		Sometimes	45	15.6
		Most of the time	16	5.6
Ongoing Work–life Stress	Has continuous stress negatively impacted your physical and mental well-being after the pandemic?	Rarely	205	71.2
		Sometimes	42	14.6
		Most of the time	41	14.2
	Has your work–life balance worsened since the pandemic?	Rarely	180	62.5
		Sometimes	63	21.9
		Most of the time	45	15.6
	Are flexible work policies necessary to reduce stress at work?	Rarely	168	58.3
		Sometimes	67	23.3
		Most of the time	53	18.4
Health Effects and Burnout	Have you experienced burnout or mental health issues as a result of the pandemic?	Rarely	189	65.6
		Sometimes	67	23.2
		Most of the time	32	11.1
	Has the psychological support provided by your institution been sufficient to address the stress experienced?	Rarely	202	70.1
		Sometimes	63	21.9
		Most of the time	23	8
	Are workplace wellness programs necessary to prevent burnout?	Rarely	211	73.3
		Sometimes	65	22.6
		Most of the time	12	4.2

**Table 3:** Coping mechanisms and suggestions for the future.

Coping Mechanisms and Suggestions for the Future			*n*	%
Coping Mechanisms and Self-Care	Have you used physical exercises and recreational activities to reduce stress after the pandemic?	Rarely	197	68.4
		Sometimes	43	14.9
		Most of the time	48	16.7
	Has the support group of colleagues helped you cope with daily challenges?	Rarely	206	71.5
		Sometimes	43	14.9
		Most of the time	39	13.5
	Have time off and leisure policies improved and maintained your health as a nurse?	Rarely	226	78.5
		Sometimes	41	14.2
		Most of the time	21	7.3
Perspectives and Suggestions for the Future	Has the pandemic highlighted the importance of improving working conditions for nurses?	Rarely	192	66.7
		Sometimes	37	12.8
		Most of the time	59	20.5
	Do you consider investment in training and professional development essential for the future careers of nurses?	Rarely	230	79.9
		Sometimes	53	18.4
		Most of the time	5	1.7
	Has the development of policies for psychological support been a priority in your healthcare institution?	Rarely	253	87.8
		Sometimes	30	10.4
		Most of the time	5	1.7

**Table 4:** +Socio-demographic characteristics of nurses participating in reflective seminar (*n* = 47).

Category	Subcategory	Frequency (*n*)	Percentage (%)	Mean (±SD)
Gender	Female	38	80.9	-
	Male	9	19.1	-
Age		-	-	34 (±3.4)
Hospital	Regional Hospital of Vlora	28	59.6	-
	Regional Hospital of Fier	19	40.4	-

**Table 5:** Post-COVID-18 reflections and SWOT analysis.

Category	Content	Numeric Data
Strengths	Positive coping mechanisms (self-care, physical activity, family support). - Solidarity and emotional support among staff. - Presence of ongoing training.	23 out of 47 (48.9%) have attended ongoing training.
Weaknesses	- Lack of structures for emotional recovery. - Overwork and lack of breaks. - Little training on mental health and resilience. - Lack of institutional policies for emotional care.	9 out of 47 (19.1%) had mental health training. Twelve out of 47 (25.5%) participants received resilience training.
Opportunities	- Creation of psychological support structures in the workplace. - Increasing institutional awareness. - Implementation of programs for personal development, leadership, and stress management.	15 out of 47 (33.3%) reported new healthcare programs.
Threats	- Chronic burnout and constant fatigue. - Possible departure from the profession. - Decline in the quality of patient care. - Feeling of institutional neglect.	38 of 47 (80%) had no leadership training. 33 of 47 (70.2%) had no crisis training.

**Table 6:** Interpretation of results for the pre- and post-seminar questionnaire.

	Knowledge	Before	After
1	Coping strategies	12/25.5%	20/42.6%
2	Impacts on mental health	13/27.7%	33/70.2%
3	Practical solutions	15/31.9%	36/76.6%
4	Current issues	14/29.8%	30/63.8%
5	Contribution to the profession	13/27.7%	35/74.5%
6	Lack of support	12/25.5%	18/38.3%
7	Work–life balance	13/27.7%	25/53.2%
8	Coping strategies	11/23.4%	22/46.8%

The pre-and post-intervention assessment was conducted using a structured, closed-ended questionnaire administered to all participants immediately before and after the educational seminar. The aim was to measure short-term changes in knowledge and awareness regarding post-pandemic challenges.

## Data Availability

The authors will make the raw data supporting this article’s conclusions available upon request.

## Abbreviations

The following abbreviations are used in this manuscript:

COVID-19	Corona virus Disease 2019
SWOT	Strengths, Weaknesses, Opportunities, Threats
